# FinTech revolution in mineral management: Exploring the nexus between technology adoption and sustainable Resource utilization in an industry 4.0 context

**DOI:** 10.1016/j.heliyon.2024.e24641

**Published:** 2024-01-19

**Authors:** Yuanyuan Xu, Abdelmohsen A. Nassani, Muhammad Moinuddin Qazi Abro, Imran Naseem, Khalid Zaman

**Affiliations:** a Changchun Sci-Tech University, Employment Guidance Center, Changchun, 130600, China; b Department of Management, College of Business Administration, King Saud University, P.O. Box 71115, Riyadh, 11587, Saudi Arabia; c Mehran University Institute of Science, Technology and Development, Mehran University of Engineering and Technology, Jamshoro, Sindh, Pakistan; d Department of Pakistan Studies and International Relations, Abbottabad University of Science and Technology, Abbottabad, Pakistan; e Department of Economics, The University of Haripur, Haripur, Khyber Pakhtunkhwa, 22620, Pakistan

**Keywords:** Sustainable mineral management, Financial technology, Environmental regulations, Technological readiness, Community acceptance, Australia

## Abstract

This study investigates the impact of FinTech adoption on sustainable mineral management policies in Australia within the context of Industry 4.0, using quarterly data from 1990Q1 to 2022Q4. Employing the ARDL-Bounds testing approach, Granger causality analysis, and innovation accounting matrix, the research finds a short-term positive association between FinTech adoption, technological readiness, and green mineral extraction. However, both in the short and long run, investment in sustainable mining technologies, government support for FinTech in mining, and environmental compliance exhibit a negative relationship with resource management. Bidirectional causality is observed between regulatory support for mining FinTech, technological finance solutions, and environmentally conscious mineral practices, while unidirectional causality exists from FinTech adoption to sustainable mining practices. Impulse response functions offer insights into the future impact of variables on eco-conscious mining policies, indicating positive influences from FinTech adoption, government support for FinTech in mining, and technological adaptability over the next decade. Conversely, eco-friendly mining investments, environmental conformity, and social license to operate will impact sustainable mineral utilization. Variance decomposition analysis highlights the most significant shocks on eco-friendly resource management over the next ten years, emphasizing the role of sustainable mining technologies, FinTech adoption, and public support for mining endeavours. In the transition to Industry 4.0, this research provides crucial insights for responsibly utilizing Australia's natural resources by leveraging financial technology and technological readiness.

## Highlights

• To explore the role of FinTech in sustainable mineral management in Australia's Industry 4.0.
• FinTech adoption and tech infrastructure linked to green mineral extraction.
• Sustainable mining tech investment, FinTech support, and compliance affect resource management.
• Bidirectional causality between FinTech regulations and eco-conscious practices.
• FinTech, government support, and tech readiness drive positive mineral utilization.

## 1 Introduction

In the era of Industry 4.0, the global landscape is witnessing unprecedented technological advancements, particularly in the financial sector. The rapid rise of Financial Technology (FinTech) has revolutionized traditional financial services and opened new avenues for businesses and consumers alike [1]. While its impact on the financial realm is well-documented, the implications of FinTech adoption extend far beyond the confines of finance, reaching critical sectors such as sustainable mineral management and the natural resources market [2]. The sustainable management of minerals and natural resources has become a pressing concern in the face of growing environmental challenges and the increasing demand for these valuable assets. As nations seek to balance economic growth and environmental preservation, the effective utilization and conservation of mineral resources have emerged as pivotal focal points for policymakers and industry stakeholders [3]. It is within this dynamic context that the role of FinTech in shaping sustainable mineral management policies gains prominence. This study embarks on a comprehensive exploration of the intricate relationship between FinTech adoption and sustainable mineral management policies. By examining the interplay of these two seemingly distinct domains, we aim to shed light on the potential synergies and divergences that may arise as Industry 4.0 transforms the natural resources market. The primary objective is to assess whether FinTech's incorporation can enhance the efficacy and implementation of sustainable mineral management policies, thereby contributing to a more sustainable and resilient global economy.

As we delve into this multifaceted investigation, several key aspects emerge. First and foremost, understanding the technological advancements within the FinTech sphere and their applications in the mineral industry will be crucial in grasping the opportunities and challenges that lie ahead. Whether through block chain-enabled traceability solutions, artificial intelligence-driven predictive analytics, or digitized supply chain platforms, the potential avenues for FinTech's intervention in sustainable mineral management are abundant [4]. Furthermore, the implications of FinTech adoption on the stakeholders of the natural resources market demand thorough scrutiny. From governments and regulatory bodies to mining corporations, local communities, and environmental organizations, diverse actors hold vested interests in mineral management policies. Analyzing how FinTech can mediate and augment their roles in this landscape is paramount to fostering collaboration and devising holistic approaches towards sustainable mineral utilization. Equally important is examining potential risks and drawbacks associated with integrating FinTech in mineral management [5]. While technological innovations offer unprecedented opportunities, they may also introduce novel vulnerabilities, such as data privacy concerns, cyber threats, and inequalities in access to technological resources [6]. Understanding and mitigating these challenges will be vital in harnessing the truly transformative potential of FinTech in the pursuit of sustainability. The confluence of FinTech and sustainable mineral management presents an unprecedented opportunity to foster a symbiotic relationship between technology-driven financial innovation and responsible resource utilization [7]. By unravelling the intricacies of this intersection, this research endeavors to contribute to the burgeoning discourse surrounding Industry 4.0 and sustainability, offering insights that can shape informed decisions for stakeholders across the public and private sectors alike.

In the vast landscapes of Australia, renowned for its rich and diverse natural resources, the confluence of FinTech adoption and sustainable mineral management policies has emerged as a pivotal focal point within the context of Industry 4.0 [8]. As the world undergoes a profound technological transformation, the adoption of Financial Technology (FinTech) in the Australian mineral industry holds the promise of revolutionizing traditional practices and reshaping the natural resources market in unprecedented ways. Australia's mineral wealth has long been a cornerstone of its economic growth, fueling industries and supporting global supply chains. However, in the face of mounting environmental challenges and growing concerns over resource depletion, the sustainable management of minerals has taken centre stage in national and international policy agendas [9]. Acknowledging this pressing need, Australian policymakers are increasingly turning to innovative solutions, and FinTech stands at the forefront as a potential game-changer. The integration of FinTech in sustainable mineral management policies offers many opportunities to enhance efficiency, transparency, and accountability in the mineral supply chain. Utilizing block chain technology can enable secure and immutable tracking of mineral origins, promoting responsible sourcing practices and reducing the risk of illegal mining or unethical trading [10]. Moreover, leveraging artificial intelligence and big data analytics can provide valuable insights into mineral exploration and extraction, optimizing resource allocation and minimizing environmental impact. As Australia aims to strike a harmonious balance between economic prosperity and environmental stewardship, the implications of FinTech adoption on the natural resources market loom large. With the advent of Industry 4.0 technologies, traditional mining, processing, and distribution practices are undergoing rapid transformation. Automation, IoT-enabled monitoring, and advanced robotics are reshaping operational efficiency, safety, and productivity in the sector [11]. FinTech's integration into this landscape complements these advancements by streamlining financial operations, facilitating investment in sustainable initiatives, and providing innovative financing mechanisms for responsible mining practices. However, the path to FinTech-driven sustainable mineral management is challenging. As technological disruptions unfold, questions regarding data security, privacy, and ethical considerations arise [12]. With sensitive data becoming increasingly integral to decision-making processes, safeguarding against cyber threats and ensuring responsible data usage become paramount concerns. Additionally, ensuring equitable access to FinTech solutions across all players in the natural resources market remains essential to avoid exacerbating existing disparities [13].

This study endeavors to shed light on the dynamic interplay between FinTech adoption, sustainable mineral management policies, and the natural resources market in Australia within the context of Industry 4.0. We seek to provide valuable insights for policymakers, industry stakeholders, and environmental advocates by analyzing the current landscape, opportunities, challenges, and implications. Through empirical research, we aim to contribute to the ongoing dialogue surrounding sustainable resource utilization and the transformative potential of FinTech. The fusion of FinTech and sustainable mineral management in Australia holds significant promise as a driver of positive change in the natural resources sector. By leveraging the transformative power of Industry 4.0 technologies, Australia can position itself at the forefront of responsible mineral utilization, setting new benchmarks for sustainable practices while bolstering its economic growth. As we embark on this exploration, it is essential to consider the collaborative efforts required from all stakeholders to harness the full potential of FinTech and create a thriving, equitable, and ecologically conscious natural resources market for generations to come.

Based on the stated discussion, the following research questions aim to address. First, ***how does adopting FinTech impact the efficiency and transparency of sustainable mineral management policies in Australia's natural resources market?*** With the rise of Industry 4.0 technologies, adopting FinTech solutions in Australia's natural resources sector has the potential to transform sustainable mineral management policies. Blockchain technology offers secure and immutable data recording, enabling the traceability of minerals throughout the supply chain [14]. By analyzing the utilization of FinTech applications, such as block chain and data analytics, this research aims to quantify how these innovations enhance efficiency and transparency in tracking mineral origins, ensuring responsible sourcing, and reducing the risk of illegal mining and unethical trading practices. Second, ***what are the socio-economic implications of integrating FinTech in sustainable mineral management policies in Industry 4.0?*** As Australia endeavors to balance economic growth and environmental preservation, integrating FinTech in sustainable mineral management policies can have far-reaching socio-economic consequences. By examining the impacts on various stakeholders, including mining companies, local communities, governmental agencies, and environmental organizations, this research seeks to identify potential benefits and challenges. Increased transparency in mineral supply chains may foster consumer trust and create opportunities for responsible mining operations, while technological disparities may raise concerns about equitable access to FinTech solutions [15]. Understanding these implications will aid in formulating strategies that promote inclusive and socially responsible mineral management practices in Australia. Third, ***how can FinTech-driven financing mechanisms facilitate investments in environmentally responsible mining practices and sustainable mineral exploration in Australia?*** With the mineral industry's growing focus on sustainability, innovative financing models powered by FinTech are emerging as potential catalysts for environmentally responsible mining practices and sustainable mineral exploration. By examining the feasibility and effectiveness of FinTech-driven financing mechanisms, such as crowd funding platforms, green bonds, and impact investment instruments, this research aims to explore how they can attract private and public funding for sustainable mineral initiatives. Through this analysis, policymakers and industry stakeholders can gain valuable insights into the potential role of FinTech in mobilizing financial resources to support the development and implementation of environmentally conscious mining practices in Australia.

The study has the following research objectives, help to move forward towards sustainable mineral resource extraction within the industry 4.0 adoption, i.e..

1. To assess the impact of FinTech adoption on the efficiency and transparency of sustainable mineral management policies in Australia's natural resources market.
2. To investigate the societal and economic effects of introducing FinTech into sustainable mineral management strategies and to comprehend the diverse viewpoints of participants in Australia's natural resources industry.
3. To investigate the pros and cons of using FinTech-driven funding methods to promote environmentally responsible mining practices and sustainable mineral exploration in Australia.

To better understand how private and public investments might be drawn to environmentally aware mineral exploration and extraction projects in Australia, this research aims to give policymakers and industry stakeholder's practical insights.

## 2 Literature review

A key focus topic within the framework of Industry 4.0 in recent years has been the meeting of Financial Technology (FinTech) with sustainable mineral management regulations. Integration of FinTech solutions offers a possible route for transformational change in Australia's natural resources industry, which struggles to balance economic development and ethical resource consumption in the face of mounting environmental issues. This literature review aims to examine and summarize the current body of work on how the widespread adoption of FinTech affects sustainable mineral management regulations and what that means for the natural resources industry as a whole.

### 2.1 Adoption of financial technology for mineral resource sustainability

There has been a seismic shift in the natural resources industry thanks to the application of FinTech solutions to the problem of sustainable mineral management. FinTech offers innovative tools and technologies that hold the potential to revolutionize traditional mineral supply chain practices, promoting greater efficiency and transparency. From blockchain-enabled traceability to data analytics-driven insights, the adoption of FinTech in sustainable mineral management aims to enhance responsible sourcing practices and reduce environmental impact. In a study focusing on disruptive technologies, Almansour [16] investigated the revolutionary effects of FinTech on the provision of financial services. The impact of artificial intelligence on human and nonhuman natural resources (such as minerals) available to and used by early-stage financial technology companies is discussed. Positive results are shown by qualitative data from UK FinTech workers, demonstrating how AI application improves the efficiency of operations and digital marketing. Financial inclusion in Zimbabwe's banking and financial services sectors was studied by Nyagadza et al. [17], who looked at the impact of the mobile financial technology ecosystem on the industry. The research emphasizes the importance of the environment to long-term economic growth. It stresses the need for innovative survival strategies, including digital financial technologies and the smooth use of mobile financial technology. Using panel data from 30 provinces in China, Taghizadeh-Hesary et al. [18] analyzed the effect of FinTech on the expansion of renewable energy in the country. It demonstrates that FinTech has a favourable influence on the expansion of renewable energy, with the effect being more pronounced in provinces with less expansion. The research also investigates how the Internet economy may facilitate the development of green technologies and the expansion of renewable energy sources. The results provide useful guidelines for authorities looking to encourage the use of FinTech in the renewable energy industry. The relationship between green technologies, FinTech, and natural resources in the BRICS countries was studied by Lisha et al. [19]. The research shows that across the board, detrimental effects on environmental sustainability may be attributed to FinTech and natural resources. On the other side, green innovation and economic growth benefit environmental sustainability. The results have real-world policy implications for advancing environmentally friendly technologies and energies. The work by Tiwari et al. [20] was to clarify the connection between green energy and green technology, visitors' behaviour intentions, and digital payment methods. The findings show that green energy and value perception substantially affect visitors' confidence in using digital payment methods. The convenience of digital payment methods is a major factor in the adoption of green technology and the happiness of tourists. The research provides innovative policy implications for creating long-term value in digital payments and M-wallets by increasing trust and happiness via green technology and energy.

Human resource management (HRM) and environmentally friendly innovations are the subject of research by Yang et al. [21]. Green HRM has been shown to significantly improve market capitalization agility, as shown by the findings. The research shows that enterprises and firms may improve performance by adopting flexible digitalization and human capital strategies. Financial inclusion and the proliferation of Fintech companies in India are the subjects of Asif et al. [22]. Based on these results, it is clear that providing financial services to underbanked sectors is crucial to achieving the goal of full financial inclusion, especially for the middle class. Su et al. [23] analyze the impact of natural resources on BRICS countries' economic development and sustainability from 1988 to 2021. The research shows that the rents from minerals and other natural resources promote sustainable commerce, but the rents from oil have the opposite effect. Sustainable commerce is impacted positively by digitalization and economic progress but adversely by the cost of renewable energy. The findings may be used to improve economic performance in countries with abundant natural resources. Natural resource use, carbon emission pricing, and financial sector effects of China's green economic growth are studied by Ruan et al. [24]. The research shows that the price of carbon emissions has a detrimental impact on China's green economy. In contrast, rising natural resource availability and thriving financial markets bode well for the country's green economy now and in the future. The report highlights the need to take effective steps to improve China's green economic growth by carefully managing natural resources and advancing the country's financial sector. The impact of the regional trade agreement on sustainable investment in mineral resources among the 10 ASEAN states is examined by Dong et al. [25]. The empirical estimates show that GDP negatively impacts the short term. The growth of the capital market has a beneficial effect on green investments in natural resources, both in the long and short run. Physical distance could improve green investment in mineral resources in the short and long term. The research emphasizes the value of ASEAN trade agreements and investment facilitation frameworks in attracting long-term investors to ASEAN's natural resources.

Based on the stated literature review, the study's first research hypothesis is as follows:

H1 Integrating FinTech solutions will enhance the traceability and transparency of mineral supply chains, promoting responsible sourcing practices and reducing the risk of unethical trading and illegal mining.

### 2.2 Socio-economic implications of FinTech integration in natural resources within the framework of industry 4.0

The socio-economic implications of FinTech integration in natural resources within the Industry 4.0 framework are multi-faceted. When harnessed responsibly, these technologies can drive sustainable development, empower communities, and optimize resource management, contributing to a more inclusive and environmentally conscious global economy. To further understand how technological progress and the Industry 4.0 paradigm interact, Cannavacciuolo et al. [26] undertook a comprehensive literature study. The research summarizes the current scientific literature and classifies it into several groups. The content analysis demonstrates how most published works concentrate on technical advancements in the Industry 4.0 framework. The study also provides a research agenda to guide future studies on Industry 5.0. Morawiec [27] studied how technical, organizational, and environmental variables affected enterprise resource planning software adoption and evolution. This research outlines the technologies of Industry 4.0 and highlights the need for organizational agility for businesses. Both academics and professionals may benefit from the study's findings, which provide guidelines for applying Industry 4.0 techniques and boosting organizational agility. The humanization of FinTech in the AI economy is investigated by Kazachenok et al. [28]. This research delves into the whys and hows of how blockchain might help humanize FinTech. This research adds to the literature and has real-world consequences for economic policy and development at the state level by proposing a legal and economic strategy for humanizing FinTech. Using a comprehensive literature analysis, Yaqub & Alsabban [29] examine Industry 4.0 technologies as primary agents of digital change in manufacturing. The research focuses on how these technologies improve business practices in the digital era, including competitiveness, sustainability, corporate development, and other areas. The study also discusses the difficulties of implementing Industry 4.0 and provides solutions to these problems.

In light of Industry, 4.0, AlMalki & Durugbo [30] stress the need to rethink education and expand human capability. Using a Delphi technique, this research identifies key institutional facilitators and impediments of Industry 4.0 for education, yielding national education reform suggestions. Energy transition in Belt and Road Initiative partner nations is favourably impacted by FinTech, globalization, and environmental taxation, according to research by Ullah et al. [31]. Policy implications are offered to facilitate the energy transition in various clusters of countries. In their study, Petrov et al. [32] examine the growth of green finance systems at the national level around the globe and emphasize the impact of digital technologies in this space. In order to create a conducive climate for green Fintech businesses, the study recommends that governments think about national financial intermediation models. Financial technology is investigated by Firdousi et al. [33] for its potential to increase the use of renewable energy sources and decrease carbon emissions in developing nations. The findings show FinTech's importance as deterrence against environmental degradation. Green total factor energy efficiency (GTFEE) is studied by Zhu et al. [34], who discovered a favourable association between ICT and GTFEE. We also investigate regional variation, the moderating roles played by energy structure, and the intensity of investment in R&D. The research presents data that may be used to hasten China's transition to a green economy and promote sustainable growth.

Based on the cited literature, the study's second research hypothesis is as follows:

H2 The adoption of FinTech in sustainable mineral management policies will positively impact various stakeholders in the natural resources sector, including mining corporations, local communities, government agencies, and environmental organizations.

### 2.3 FinTech-driven financing mechanisms for sustainable mineral exploration

FinTech-driven financing mechanisms are playing a crucial role in revolutionizing the landscape of sustainable mineral exploration. Traditional financing methods for mining projects often faced challenges related to transparency, accessibility, and inclusivity. However, with the integration of innovative FinTech solutions, such as blockchain-based crowdfunding platforms, digital tokens, and smart contracts, new opportunities are emerging for sustainable mineral exploration projects. Milian et al. [35] conducted a Systematic Literature Review to explore the concept of fintech and map the literature in the field. The findings light the many different ways that Fintech may be defined and used, with particular attention paid to financial services, innovations, and regulation. The study by Suryono et al. [36] aimed to assess the current status of research on financial technology, identify research gaps, and investigate potential future research challenges and trends. The research classified Fintech services and highlighted the sharing economy, laws, and IT as key motivators. Cao et al. [37] communicated about the development of Smart FinTech, which uses DSAI methods to power smart and automated financial enterprises and services. The study provided a synopsis of DSAI applications throughout the FinTech industry. The major facilitators of FinTech innovation in Saudi Arabia were studied by Makki et al. [38], who used interpretative structural modelling and an analytic network approach in their investigation. Researchers hoped their findings would help governments and industry practitioners promote FinTech expansion. Kao et al. [39] created a hybrid decision model for financial institutions to facilitate technological adoption in supply chain financing. The research compared several Fintech approaches and emphasized the benefits of blockchain-based alternatives. The results were meant to help banks create supply chain finance businesses using Fintech.

Zeng [40] examined the climate change mitigation efforts of Ant Forest, a gamified green project founded by a Chinese Fintech firm. Researchers identified a discrepancy between the environmental benefit of Ant Forest and its effect on consumer behaviour. As a capitalist green effort, Ant Forest followed the logic of competition and growth by depending on market forces and consumer demand. It also led to a disconnection between people and the environmental impacts of their consumption habits, creating a knowledge gap. Munodei & Sibindi [41] did a bibliometric study investigating the connections between financial technology and welfare programs. According to the findings, research into social security has begun to concentrate more on Fintech since 2018. Fintech as a buffer against climate change effects, blockchain technology research in social protection, Fintech in health care service provision, and other related topics were highlighted as emergent research areas. Two experimental tests were undertaken by Gonçalves et al. [42] with FinTech customers to examine how they felt about receiving credit judgments from AIs instead of humans. Research shows that customers' reactions to AI credit choices vary by product. Personal loan applicants turned down by an AI service reported greater levels of satisfaction than those whom a credit analyst had turned down. This study adds to our knowledge of how customers feel about FinTech companies using AI. Asif et al. [43] emphasized several types of risks in the monetary technology sector's digital environment. They looked at 719 papers that were relevant to the topic of technology hazards in banking as they relate to FinTech. Cyber security risk, data theft, financial crimes, operational risk, default risk, money laundering, and financial terrorism are only some risk categories the research highlighted in the context of FinTech and the banking industry.

Based on the stated literature, the study's final hypothesis is as follows:

H3 FinTech-driven financing mechanisms will effectively mobilize financial resources for sustainable mineral exploration projects.

Several gaps in the earlier study's coverage warrant further investigation to gain a more comprehensive understanding of the subject matter. One of the crucial missing gaps lies in the technological readiness and adoption rate of FinTech solutions in the Australian natural resources market. While the study acknowledged the potential transformative power of FinTech, it did not delve into the current state of adoption among mining companies and other stakeholders. Understanding the extent to which these technologies have been embraced is essential in assessing their overall impact on sustainable mineral management policies [44]. The potential for future integration and enhancement may be better understood by investigating these solutions' technical maturity and readiness. The natural resources industry is another crucial area that needs further research on the environmental effect of FinTech adoption. The preceding research should have included an in-depth investigation of the ecological footprint associated with applying these technologies [45,46], although acknowledging the potential for FinTech to improve sustainability practices. Using technologies that promote environmental sustainability requires an environmental impact evaluation of FinTech solutions. Policymakers and industry stakeholders may prioritize green and sustainable practices by examining these technologies' carbon footprint, resource usage, and possible dangers. A thorough investigation of the legal and regulatory frameworks controlling FinTech's incorporation into the Australian natural resources industry should have been included in the prior research [47,48]. Complying with applicable environmental and data protection standards requires an in-depth understanding of these frameworks and identifying possible hurdles or facilitators to FinTech adoption. The study may help guide the prudent and ethical integration of FinTech solutions in the natural resources industry by providing insight into the current regulatory context. Furthermore, the prior research [49,50] could have effectively addressed stakeholder perspectives and issues linked to FinTech adoption in sustainable mineral management. Understanding the perspectives and issues of mining firms, local people, government agencies, and environmental groups is crucial for building trust and working together. By understanding potential resistance to change and addressing stakeholders' concerns, the research can contribute to a more holistic approach to sustainable mineral management that incorporates diverse perspectives and promotes shared decision-making. In light of these missing gaps, the contribution of this study lies in its comprehensive assessment of the impact of FinTech adoption on sustainable mineral management policies in Australia. By addressing the gaps in the earlier research, this study enriches the existing knowledge and provides a more nuanced understanding of the complex relationship between FinTech and sustainable mineral management. Through rigorous evaluations of technological readiness, environmental considerations, regulatory landscapes, and stakeholder engagement, the research offers valuable insights for policymakers, mining companies, investors, and environmental advocates. The study's findings provide a roadmap for leveraging FinTech's potential to drive positive transformations in sustainable mineral management policies while preserving the ecological integrity of Australia's natural resources. Ultimately, the research contributes to the ongoing discourse surrounding Industry 4.0 and sustainability, offering informed guidance for promoting responsible and environmentally conscious practices in the natural resources market.

## 3 Data and methodology

The study used **Sustainable Mineral Management (SMM)** as a response variable, which refers to the responsible and efficient management of mineral resources to ensure their extraction and utilization align with environmental, social, and economic sustainability principles. It involves practices that minimize negative environmental impacts, support local communities, and promote long-term economic viability while utilizing mineral resources. For this purpose, the study used mineral depletion date (MDR, % of GNI)' that measures the rate at which mineral resources are depleted over time. It represents the ratio of mineral extraction to the remaining mineral reserves. A lower MDR indicates a more sustainable management of mineral resources, implying a slower depletion rate than the available reserves. The following list of explanatory variables is used in the study, i.e..

I. **FinTech Adoption (FinTech):** It refers to the extent to which individuals and businesses adopt financial technologies (FinTech) to access and manage financial services. FinTech encompasses many technological innovations that aim to improve and streamline financial activities, including digital payment systems, online banking, peer-to-peer lending, robo-advisors, and blockchain-based solutions. The study used internet users (% of individuals) for financial transactions, which measures the proportion of the population that uses the internet to conduct financial transactions, such as making online payments, using mobile banking apps, or engaging in other financial activities through digital platforms, within the Australian mining and natural resources sector.
II. **Investment in Sustainable Mining Technologies (ISMT):** It refers to the total amount of funding and investment directed towards developing, implementing, and utilizing sustainable mining technologies in Australia. This could include investments in renewable energy for mining operations, water recycling systems, waste management technologies, and other environmentally friendly practices. By subtracting the mineral resource rents from the R&D expenditures, the study isolates the portion of financial resources that mining companies are investing specifically in R&D activities for mining technology.
III. **Government Support for FinTech in Mining (GSFTM):** It refers to the level of encouragement, assistance, and policies the government provides to promote and facilitate the adoption of FinTech solutions within the mining industry. FinTech in mining involves the application of digital technologies to enhance financial processes, improve operational efficiency, and optimize financial management in the mining sector. The study used the 'Ease of doing business score (0 = lowest performance to 100 = best performance)', which assesses the ease of conducting business activities in a country. Higher scores may suggest a favourable business environment, influencing government support for innovation, including FinTech adoption in mining.
IV. **Environmental Compliance (ENVCOMP):** It measures the level of compliance of mining companies with environmental regulations and standards. This variable reflects the industry's commitment to environmentally responsible practices. The study used CO2 emissions from manufacturing industries and construction (% of total fuel combustion) to assess the mining industry's environmental impact and compliance with emissions regulations. A lower percentage suggests that the mining industry effectively manages and reduces its carbon emissions from manufacturing and construction activities, indicating a more environmentally responsible approach. Conversely, a higher percentage may indicate higher emissions and potentially lower compliance with emissions regulations.
V. **Social License to Operate (SLO) Index**: A composite index that gauges the acceptance and support of local communities for mining operations in Australia. A higher SLO score indicates positive engagement and cooperation between mining companies and affected communities. The study used the Rule of Law Index (−2.5 to 2.5), which assesses the extent to which a country's rule of law is upheld. It can indirectly reflect the legal framework and protection of community rights related to mining activities.
VI. **Technological Infrastructure Readiness (TIR, per 100 individuals):** It assesses the readiness of technological infrastructure in Australia to support FinTech adoption within the mining industry. This includes access to high-speed internet, availability of data centers, and overall digital connectivity. The study used Fixed Broadband Subscriptions, which variable represents the number of fixed broadband subscriptions per 100 people. It reflects the access to high-speed internet, which is essential for adopting FinTech solutions in the mining industry.

The data for the variables used in this study were sourced from the World Development Indicators database [51]. The quarterly dataset spans the years 1990Q1 through 2022Q4 and is focused on the economic climate of Australia. Table 1 lists all of the variables considered in the study, along with detailed descriptions of each factor.

**Table 1:** List of variables and measurement.

Variables	Symbol	Indicator	Measurement	Justification
Sustainable Mineral Management	SMM	Mineral Depletion Rate (% of GNI)	SMM (%) = (Mineral Extraction/Remaining Mineral Reserves) * 100	Indicator of responsible mineral resource management, considering extraction rate and remaining reserves.
FinTech Adoption	FinTech	Internet Users (% of individuals)	FinTech Adoption (%) = (Number of Individuals Using the Internet for Financial Transactions/Total Population) * 100	Measure of FinTech adoption in mining sector based on internet usage for financial transactions.
Investment in Sustainable Mining Technologies	ISMT	R&D Expenditures (% of GDP) - Mineral Resource Rents (% of GDP)	ISMT (% of GDP) = R&D Expenditures (% of GDP) - Mineral Resource Rents (% of GDP)	Calculation of investments in sustainable mining technologies, accounting for mineral resource rents.
Government Support for FinTech in Mining	GSFTM	Ease of Doing Business Score (0–100)	Ease of Doing Business Score (0–100) = (Σ(Indicator Score * Indicator Weight)) * (100/Maximum Possible Score)	Score reflecting government support for FinTech adoption in mining industry based on ease of doing business.
Environmental Compliance	ENVCOMP	CO2 emissions from manufacturing industries and construction (% of total fuel combustion)	ENVCOMP (%) = (CO2 emissions/total fuel combustion) * 100	Indicator of mining companies' environmental compliance based on CO2 emissions from manufacturing and construction.
Social License to Operate	SLO	Rule of Law Index (−2.5 to 2.5)	SLO = ((Index Value - 0)/(100–0)) * (2.5 - (−2.5)) + (−2.5)	Measure of social license to operate based on the transformed Rule of Law Index.
Technological Infrastructure Readiness	TIR	Fixed Broadband Subscriptions per 100 individuals	TIR = (Number of Fixed Broadband Subscriptions/Total Population) * 100	Indicator of technological infrastructure readiness for FinTech adoption in mining based on fixed broadband subscriptions.

Australia's significance as an urbane economy with a notable emphasis on FinTech adoption and its substantial position in the natural resources industry justifies the nation's choice and the period from 1990 to 2022 for the study's research. Moreover, there are numerous justifications for this. To begin, Australia is a perfect case study for examining the effects of FinTech adoption because of its mature economy, advanced financial sector, and significant focus on technical improvements [52]. Since Australia has been an early adopter of digital transformation and FinTech technologies, it is a fitting location to investigate how these developments could affect mineral management strategies for long-term sustainability. Second, Australia's natural resources industry is crucial. The mining industry is crucial to the country's economy because of its abundant natural resources [53]. As the mining sector faces new issues brought on by resource extraction and environmental concerns, its leaders must have a firm grasp on how the widespread use of FinTech impacts regulations for the responsible management of these minerals. This research has the potential to serve as a template for other resource-rich nations struggling with comparable issues by illuminating the interplay between FinTech adoption and Australia's natural resources industry via the lens of Australia. The adoption of FinTech and changes in sustainable mineral management practices are examined in depth during 1990Q1–2022Q4. The timeline covers many historical moments, from the birth of the internet to the rise of financial technology to the birth of Industry 4.0 ideas. By looking at this time frame, we can see how the adoption of FinTech coincided with changes in mineral management regulations and how this fits into the larger context of technological development and industrial transformation.

### 3.1 Theoretical framework

The theory "disruptive innovation" was invented by Clayton Christensen to describe how new technology or business models may shake up established markets and sectors [54]. In light of the study's overarching goal of elucidating FinTech's potential for bringing about profound changes in the mining industry, the Disruptive Innovation Theory becomes an important lens through which to view these developments—the mining sector benefits greatly from introducing disruptive technologies made possible by the use of FinTech. Companies in the mining industry may improve productivity, save costs, and optimize resource management using cutting-edge technology like data analytics and automation [55]. This FinTech-driven revolution in the mining industry may significantly impact sustainable mineral management policies. Moreover, FinTech adoption can create new market opportunities in the mining sector. As innovative FinTech applications emerge, new business models and services may develop. FinTech could enable crowdfunding for sustainable mining projects or create new investment opportunities that align with environmental and social criteria [56]. These novel opportunities can stimulate interest in sustainable mineral management practices and attract stakeholders who prioritize ESG principles. However, with disruptive innovation comes challenges for incumbent mining companies. Failing to embrace FinTech solutions may face competitive disadvantages, losing market share to more agile and tech-savvy players. The study can delve into how the adoption of FinTech affects the competitive dynamics in the mining industry and how established companies respond to the disruptive forces unleashed by technology. In the broader context, adopting FinTech can lead to transforming the entire mining industry. FinTech-driven transparency and traceability solutions could reshape supply chain practices, encouraging responsible sourcing and sustainability throughout the mining sector [57]. The theoretical underpinnings of Industry 4.0 and the circular economy within Global Value Chains are necessary to understand the complex link between technological advancement and environmental responsibility. Awan et al. [58] examine how Industry 4.0 might include IoT in circular economy management. This study illuminates significant stakeholders' interests, adding to the theoretical framework. The study gives a theoretical lens to explore the complex dynamics of circular economy development in Industry 4.0, including its risks and advantages. Based on this theory, Awan et al. [1] propose a Global Value Chain method that theoretically examines Industry 4.0, circular economy practises, and GVCs. The research carefully evaluates 112 peer-reviewed papers to show how Industry 4.0 technology and circular economy practises in GVCs are interconnected. This improves our theoretical understanding and shows GVC research's limits, particularly where Industry 4.0 and the circular economy converge. The study can assess the implications of FinTech adoption on the structure and operations of the mining industry, understanding how it influences the evolution of sustainable mineral management policies. By examining the mining sector through the lens of the Disruptive Innovation Theory, researchers can gain insights into how FinTech has the potential to revolutionize traditional mining practices, create new opportunities, challenge incumbents, and contribute to the advancement of sustainable mineral management in Australia. Understanding the transformative power of FinTech in the mining industry can shed light on the future trajectory of the sector in the context of Industry 4.0 and its implications for the natural resources market.

### 3.2 Econometric framework

#### 3.2.1 Unit root test

The data set underwent a series of sequential statistical techniques to obtain parameter estimates and formulate sustainable policies. The first step involved conducting a Unit Root Test, which used an autoregressive component to analyze the time-varying stationary nature of the variables of interest. The AR(1) model was employed for this purpose, expressed in Equation (1):

(1) $$\varepsilon_{t}=\alpha +\beta_{t}$$

In this equation, ε_t_ represents the error term. The Unit Root test helped determine the integration order of the candidate variables and assess their stationarity characteristics.

There were four possible outcomes from the Unit Root Test:

1. In Case 1, if β = 0, the series was identified as level stationary.
2. In Case 2, if β = 1, the series was found to explode.
3. In Case 3, if β > 1, the variable was deemed non-stationary.
4. In Case 4, if β < 1, the series was considered differenced stationary.

The study group determined the proper differencing or transformation required to render the series stationary using the Augmented Dickey-Fuller (ADF) unit root test with the equation above. This was a necessary precondition to estimating parameters and confidently performing statistical analysis.

Time series analysis relies heavily on the assumption of stationarity, which guarantees that the data's statistical features remain constant throughout time. Incorrect regression findings and flawed model predictions may emerge from data that is not perfectly stationary. The ADF test looks for evidence of a unit root to determine whether a series is non-stationary and subject to a stochastic trend. Without mean reversion, a unit root in the data indicates that the series is moving randomly. However, if the data is stable, its mean and variance are unchanging.

#### 3.2.2 ARDL-bounds testing approach

The ARDL-Bounds testing method, introduced by Pesaran et al. [59], uses unit root estimation to integrate orders 0 and 1 variables. Odd outcomes may occur when using a single regression equation to analyze data with both stationary I(0) and non-stationary I(1) variables. Difference and lag operators are used to solve the simultaneous event problem. For parameter estimation in ARDL-Bounds testing, the long-term trend of the variables is known in advance. The error correction component is introduced during regression to account for the convergence of the model. Researchers may avoid the difficulties associated with mixed-order variables by using the ARDL specification to integrate the I(0) properly and I(1) variables in the regression equation. The model considers the dynamic modifications by considering the error correction term, which guarantees convergence in the long run.

The ARDL-Bounds testing method provides a solid structure for predicting the connections between the relevant variables and developing long-term solutions. The technique thoroughly evaluates the model's long-term interactions and convergence by considering both stationary and non-stationary variables and adding the error correction component. Given the changing nature of the variables and their interdependence, this approach is appropriate for the study's intended purpose of examining the effect of FinTech adoption on sustainable mineral management policy in Australia. The model estimation ARDL specification is shown in Equation (2):

(2) $$\ln\;{\left(SMM\right)}_{t}=\alpha_{0}+\sum_{i=1}^{p}\varphi_{i}\Delta \ln\;{\left(SMM\right)}_{t-i}+\sum_{i=0}^{q}\theta_{i}\Delta \ln\;{\left(FINTECH\right)}_{t-i}+\sum_{i=0}^{r}\theta_{i}\Delta \ln\;{\left(ISMT\right)}_{t-i}+\sum_{i=0}^{s}\varphi_{i}\Delta \ln\;{\left(GSFTM\right)}_{t-i}+\sum_{i=0}^{t}\varphi_{i}\Delta \ln\;{\left(ENVCOMP\right)}_{t-i}+\sum_{i=0}^{u}\varphi_{i}\Delta \ln\;{\left(SLO\right)}_{t-i}+\sum_{i=0}^{v}\varphi_{i}\Delta \ln\;{\left(TIR\right)}_{t-i}+\delta_{1}\;\ln\;{\left(FINTECH\right)}_{t}+\delta_{2}\;\ln\;{\left(ISMT\right)}_{t}+\delta_{3}\;\ln\;{\left(GSFTM\right)}_{t}+\delta_{4}\;\ln\;{\left(ENVCOMP\right)}_{t}+\delta_{5}\;\ln\;{\left(SLO\right)}_{t}+\delta_{6}\;\ln\;{\left(TIR\right)}_{t}+\rho ECT_{t-i}+\varepsilon_{t}$$

Where, Δ shows the first difference operator, and ECT shows the disturbance term.

#### 3.2.3 Granger causality

The Granger causality test was used to analyze the correlations between the variables of interest. The Granger causality test is a statistical technique for determining whether or not one variable may explain or predict shifts in a second. It aids in establishing the nature and magnitude of the relationships between the relevant factors. Granger's causality test uses the F-test to determine whether or not the variables in question have unidirectional or bidirectional causal relationships or if the strength of the statistical correlation between them renders the relationship neutral. The results of the Granger causality test greatly aid long-term growth strategies and the interplay of factors. There may be the following observable causal relationships between the variables:

I. **Unidirectional Causality**: This implies a one-way causality from Sustainable Mineral Management (SMM) to other variables, but not vice versa. In other words, SMM has a significant predictive influence on other variables, but those variables do not influence SMM in return.
II. **Reverse Causality**: In this scenario, other variables Granger cause SMM, meaning they have a predictive influence on SMM, but SMM does not influence them in return.
III. **Bidirectional Causality**: This indicates that the variables have a two-way link. SMM and other variables can predict and influence each other, forming a reciprocal relationship.
IV. **Neutrality**: The relationship is deemed neutral when variables do not confirm any causality pattern between them. This means that no statistically significant causal links are observed between the variables.

To conduct the Granger causality test, the study utilized the Vector Autoregression (VAR) framework, represented by equation (3):

(3) $$\left[\begin{array}{l} \ln\;{\left(SMM\right)}_{t}\\ \ln\;{\left(FINTECH\right)}_{t}\\ \ln\;{\left(ISMT\right)}_{t}\\ \ln\;{\left(GSFTM\right)}_{t}\\ \ln\;{\left(ENVCOMP\right)}_{t}\\ \ln\;{\left(SLO\right)}_{t} \end{array}\right]=\left[\begin{array}{l} \tau_{0}\\ \tau_{1}\\ \tau_{2}\\ \tau_{3}\\ \tau_{4}\\ \tau_{5} \end{array}\right]+\sum_{i=1}^{p}\left[\begin{array}{l} \sigma_{11t}\sigma_{12t}\sigma_{13t}\sigma_{14t}\sigma_{15t}\\ \sigma_{21t}\sigma_{22t}\sigma_{23t}\sigma_{24t}\sigma_{25t}\\ \sigma_{31t}\sigma_{32t}\sigma_{33t}\sigma_{34t}\sigma_{35t}\\ \sigma_{41t}\sigma_{42t}\sigma_{43t}\sigma_{44t}\sigma_{45t}\\ \sigma_{51t}\sigma_{52t}\sigma_{53t}\sigma_{54t}\sigma_{55t}\\ \sigma_{61t}\sigma_{62t}\sigma_{63t}\sigma_{64t}\sigma_{65t} \end{array}\right]\times \left[\begin{array}{l} \ln\;{\left(SMM\right)}_{t-1}\\ \ln\;{\left(FINTECH\right)}_{t-1}\\ \ln\;{\left(ISMT\right)}_{t-1}\\ \ln\;{\left(GSFTM\right)}_{t-1}\\ \ln\;{\left(ENVCOMP\right)}_{t-1}\\ \ln\;{\left(SLO\right)}_{t-1} \end{array}\right]+\sum_{j=p+1}^{d\;\max}\left[\begin{array}{l} \theta_{11j}\theta_{12j}\theta_{13j}\theta_{14j}\theta_{15j}\\ \theta_{21j}\theta_{22j}\theta_{23j}\theta_{24j}\theta_{25j}\\ \theta_{31j}\theta_{32j}\theta_{33j}\theta_{34j}\theta_{35j}\\ \theta_{41j}\theta_{42j}\theta_{43j}\theta_{44j}\theta_{45j}\\ \theta_{51j}\theta_{52j}\theta_{53j}\theta_{54j}\theta_{55j}\\ \theta_{61j}\theta_{62j}\theta_{63j}\theta_{64j}\theta_{65j} \end{array}\right]\times \left[\begin{array}{l} \ln\;{\left(SMM\right)}_{t-j}\\ \ln\;{\left(FINTECH\right)}_{t-j}\\ \ln\;{\left(ISMT\right)}_{t-j}\\ \ln\;{\left(GSFTM\right)}_{t-j}\\ \ln\;{\left(ENVCOMP\right)}_{t-j}\\ \ln\;{\left(SLO\right)}_{t-j} \end{array}\right]+\left[\begin{array}{l} \varepsilon_{1}\\ \varepsilon_{2}\\ \varepsilon_{3}\\ \varepsilon_{4}\\ \varepsilon_{5}\\ \varepsilon_{6} \end{array}\right]$$

Equations (4), (5), (6), (7), (8), (9), (10) shows Granger causality in multivariate system, i.e.,

(4) $$SMM_{t}=c_{1}+{\sum_{i=1}^{2}\beta_{1}SMM}_{t-i}+\sum_{i=1}^{2}\beta_{2}FINTECH_{t-i}+\sum_{i=1}^{2}\beta_{3}ISMT_{t-i}+\sum_{i=1}^{2}\beta_{4}GSFTM_{t-i}+{\sum_{i=1}^{2}\beta_{5}ENVCOMP}_{t-i}+{\sum_{i=1}^{2}\beta_{6}SLO}_{t-i}+{\sum_{i=1}^{2}\beta_{7}TIR}_{t-i}+\varepsilon$$

(5) $$FINTECH_{t}=c_{1}+{\sum_{i=1}^{2}\beta_{1}FINTECH}_{t-i}+{\sum_{i=1}^{2}\beta_{2}SMM}_{t-i}+\sum_{i=1}^{2}\beta_{3}ISMT_{t-i}+\sum_{i=1}^{2}\beta_{4}GSFTM_{t-i}+{\sum_{i=1}^{2}\beta_{5}ENVCOMP}_{t-i}+{\sum_{i=1}^{2}\beta_{6}SLO}_{t-i}+{\sum_{i=1}^{2}\beta_{7}TIR}_{t-i}+\varepsilon$$

(6) $$ISMT_{t}=c_{1}+{\sum_{i=1}^{2}\beta_{1}ISMT}_{t-i}+\sum_{i=1}^{2}\beta_{2}FINTECH_{t-i}+\sum_{i=1}^{2}\beta_{3}SMM_{t-i}+\sum_{i=1}^{2}\beta_{4}GSFTM_{t-i}+{\sum_{i=1}^{2}\beta_{5}ENVCOMP}_{t-i}+{\sum_{i=1}^{2}\beta_{6}SLO}_{t-i}+{\sum_{i=1}^{2}\beta_{7}TIR}_{t-i}+\varepsilon$$

(7) $$GSFTM_{t}=c_{1}+{\sum_{i=1}^{2}\beta_{1}GSFTM}_{t-i}+\sum_{i=1}^{2}\beta_{2}FINTECH_{t-i}+\sum_{i=1}^{2}\beta_{3}ISMT_{t-i}+\sum_{i=1}^{2}\beta_{4}SMM_{t-i}+{\sum_{i=1}^{2}\beta_{5}ENVCOMP}_{t-i}+{\sum_{i=1}^{2}\beta_{6}SLO}_{t-i}+{\sum_{i=1}^{2}\beta_{7}TIR}_{t-i}+\varepsilon$$

(8) $$ENVCOMP_{t}=c_{1}+{\sum_{i=1}^{2}\beta_{1}ENVCOMP}_{t-i}+\sum_{i=1}^{2}\beta_{2}FINTECH_{t-i}+\sum_{i=1}^{2}\beta_{3}ISMT_{t-i}+\sum_{i=1}^{2}\beta_{4}GSFTM_{t-i}+{\sum_{i=1}^{2}\beta_{5}SMM}_{t-i}+{\sum_{i=1}^{2}\beta_{6}SLO}_{t-i}+{\sum_{i=1}^{2}\beta_{7}TIR}_{t-i}+\varepsilon$$

(9) $$SLO_{t}=c_{1}+{\sum_{i=1}^{2}\beta_{1}SLO}_{t-i}+\sum_{i=1}^{2}\beta_{2}FINTECH_{t-i}+\sum_{i=1}^{2}\beta_{3}ISMT_{t-i}+\sum_{i=1}^{2}\beta_{4}GSFTM_{t-i}+{\sum_{i=1}^{2}\beta_{5}ENVCOMP}_{t-i}+{\sum_{i=1}^{2}\beta_{6}SMM}_{t-i}+{\sum_{i=1}^{2}\beta_{7}TIR}_{t-i}+\varepsilon$$

(10) $$TIR_{t}=c_{1}+{\sum_{i=1}^{2}\beta_{1}TIR}_{t-i}+\sum_{i=1}^{2}\beta_{2}FINTECH_{t-i}+\sum_{i=1}^{2}\beta_{3}ISMT_{t-i}+\sum_{i=1}^{2}\beta_{4}GSFTM_{t-i}+{\sum_{i=1}^{2}\beta_{5}ENVCOMP}_{t-i}+{\sum_{i=1}^{2}\beta_{6}SLO}_{t-i}+{\sum_{i=1}^{2}\beta_{7}SMM}_{t-i}+\varepsilon$$

If the null hypothesis is accepted, there is no connection between the independent and dependent variables. If the null inference is rejected, then there is a relationship between the two variables. This allows the model to make predictions about the relationships between the variables.

#### 3.2.4 Innovation accounting matrix (IAM)

The IAM approach comprises the following, i.e..

- **Impulse Response Function (IRF)**: The IRF is a statistical tool used to decompose the variance of the dependent variable (SMM) into the contributions of its innovations and the innovations of the independent variable. In other words, it helps us understand the impact of shocks or changes in each independent variable on the dependent variable over time. It is possible to calculate how much each autonomous variable and its lagging values contribute to the total variance in SMM by using the IRF analysis, and understanding how shocks or changes in one variable impact other variables, notably the conditional variable (SMM), is made possible by this method. Policymakers, stakeholders, and industry leaders may use this data to make choices and formulate plans that advance sustainable practices and boost the mining industry's overall performance.
- **Variance Decomposition Analysis (VDA)**: The VDA ranks the significance of each independent variable in explaining the time-varying dispersion of the dependent variable (SMM). Insights into the primary forces influencing shifts in SMM and their relative contributions to the observed variance in the dependent variable are provided by this research. Using the VDA, we can see how much of the total variation in SMM can be attributable to each independent variable (FinTech, ISMT, GSFTM, ENVCOMP, SLO, and TIR). It measures how much progress in each independent variable contributes to explaining the swings in SMM. Scientists may learn which independent factors have the greatest influence on SMM and which factors contribute comparatively less variability by using the VDA. The major causes and variables influencing sustainable mineral management strategies may be better understood with this knowledge.

## 4 Results and discussion

The distribution and features of the variables in Australia from 1990Q1 to 2022Q4 are shed light on by the descriptive statistics in Table 2. The average value of Sustainable Mineral Management (SMM) is 0.912 %. This indicates that mineral extraction in Australia has cost the country around 0.912 % of its GNI each year on average for the period under study. At a time, the mineral depletion rate was as high as 1.970 % of GNI, suggesting that resources were being heavily exploited during that age. However, the lowest documented mineral depletion rate is just 0.303 %, which reflects a period when mineral extraction was very light. The growing trend may be explained by the increased demand for minerals caused by Australia's burgeoning economy and expanding industrial sector during this time. There is a clear rising trend in the use of financial technologies (FINTECH), suggesting that more and more Australians are increasingly relying on the Internet to manage their finances. The mean figure of 55.624 % indicates that, on average, around 55.62 % of people were internet users during the time being analyzed. The maximum value of 96.394 % indicates a notable peak in internet usage, possibly reflecting increased digitalization and technological advancements. This trend can be attributed to the rapid advancement of technology and increased internet penetration during this period. The proliferation of internet connectivity and the widespread use of mobile devices has facilitated the adoption of various financial technologies, making financial services more accessible and efficient for individuals in Australia.

**Table 2:** Descriptive statistics.

Variables	SMM	FINTECH	ISMT	GSFTM	ENVCOMP	SLO	TIR
Mean	0.912	55.624	−0.810	76.772	13.252	1.718	15.410
Maximum	1.970	96.394	0.991	82.200	16.509	1.918	35.448
Minimum	0.303	0.585	−8.636	70	9.765	1.171	0.637
Std. Dev.	0.570	34.031	2.303	3.662	2.095	0.112	14.183
Skewness	0.541	−0.554	−2.402	−0.313	0.166	−3.425	0.112
Kurtosis	1.870	1.861	8.710	1.883	1.785	18.592	1.284

Investment in Sustainable Mining Technologies (ISMT) shows a mean value of −0.810 %, indicating that, on average, investment in sustainable mining technologies was approximately −0.810 % of GDP, implying that R&D expenditures were lower than mineral resource rents. Various economic factors might drive this trend. For instance, short-term profit motives, uncertainty regarding the long-term returns from sustainable technologies, or a lack of adequate incentives and regulations for sustainable mining practices might have influenced companies' investment decisions. Government Support for FinTech in Mining (GSFTM) exhibits a mean value of 76.772, indicating that, on average, the ease of doing business in the FinTech sector related to mining was relatively high in Australia. The maximum value of 82.200 suggests that there have been instances when the government's support for FinTech in mining was even higher, potentially reflecting periods of intensified efforts to promote FinTech innovation. On the other hand, the minimum value of 70 indicates a relatively low level of government support for FinTech in mining at its lowest point, possibly representing times when FinTech initiatives faced challenges or lacked strong governmental backing. Environmental Compliance (ENVCOMP) indicates a mean value of 13.252 %, suggesting that, on average, CO2 emissions from manufacturing industries and construction constituted approximately 13.25 % of total fuel combustion emissions in Australia. The standard deviation of 2.095 shows the variability in CO2 emission levels over the years. The slightly positive skewness of 0.166 suggests a nearly symmetrical distribution, indicating a relatively balanced distribution of instances with higher and lower emissions. This trend could be influenced by economic factors such as industrial growth and expansion, which often result in increased carbon emissions. However, efforts towards environmental sustainability, technological advancements in green energy, and more environmentally friendly manufacturing processes might have helped mitigate the overall impact of emissions. Social License to Operate (SLO) has a mean value of 1.718, indicating a relatively high rule of law in Australia and a stronger social license to operate for mining companies. Public perception and support for mining activities can be sensitive to changes in environmental impact, social engagement, and corporate responsibility, contributing to the fluctuations in the social license to operate. Technological Infrastructure Readiness (TIR) exhibits a mean value of 15.410, indicating that, on average, there are approximately 15.41 fixed broadband subscriptions per 100 individuals in Australia. The maximum value of 35.448 suggests that there has been relatively higher technological infrastructure readiness, potentially representing times when technological connectivity and access peaked. This upward trend in technological infrastructure readiness can be attributed to ongoing investments in telecommunication infrastructure and the advancement of technology in the country. Better technological infrastructure facilitates greater connectivity, which, in turn, supports the adoption of financial technology and sustainable mining practices and enhances overall economic productivity.

Table 3 presents the ADF unit root estimates, which reveal the stationarity properties of the variables under consideration. The results indicate that SLO and TIR are level stationary, meaning they do not require differencing to achieve stationarity. On the other hand, the remaining variables, including SMM, FINTECH, ISMT, GSFTM, and ENVCOMP, are first differenced stationary. In order to attain stationarity, it is necessary to difference these variables only once.

**Table 3:** ADF unit root estimates.

Variables	Level		First Difference		Decision
	Constant	Constant and Trend	Constant	Constant and Trend	
SMM	−1.401 (0.569)	−2.605 (0.280)	−5.951 (0.000)	−5.876 (0.000)	I(1)
FINTECH	−1.302 (0.616)	−0.634 (0.969)	−3.134 (0.034)	−3.283 (0.087)	I(1)
ISMT	0.110 (0.961)	−1.243 (0.883)	−5.526 (0.000)	−5.917 (0.000)	I(1)
GSFTM	−2.079 (0.253)	−1.580 (0.778)	−6.182 (0.000)	−6.933 (0.000)	I(1)
ENVCOMP	−1.600 (0.470)	−0.628 (0.970)	−6.033 (0.000)	−6.748 (0.000)	I(1)
SLO	−4.582 (0.000)	−4.661 (0.003)	−8.898 (0.000)	−8.764 (0.000)	I(0)
TIR^a^	−5.200 (0.000)	−5.391 (0.000)	−4.445 (0.049)	−5.597 (0.000)	I(0)

The need to estimate both short- and long-term parameters using ARDL-Bounds testing is supported by the fact that the sequence of the integrated variables is often unclear. With its ability to include stationary and non-stationary variables, the ARDL model is well-suited to studying the interrelationships between time series data points. By using the ARDL-Bounds testing method, we can better understand the interplay between these economic factors and the consequences this may have for future policy and decision-making.

Table 4 shows the estimations for the criterion for choosing the lag time. According to the outcomes, a single lag length is best for the specified model when considering LR, FPE, SIC, and HQ criteria. On the other hand, the 'AIC' criteria indicate that a lag duration of up to 2 may be used for the existing models. Our research included up to two lag durations since this maximized the 'AIC' score. This choice of lag time allows for a more complete understanding of the economic interactions and consequences of the variables by capturing possible short- and medium-term dynamics between them.

**Table 4:** Lag length criterion estimates.

Lag	LogL	LR	FPE	AIC	SC	HQ
0	−346.322	NA	18.728	22.795	23.118	22.900
1	−173.151	256.963*	0.0067*	14.783	17.374*	15.628*
2	−123.748	50.996	0.011	14.757*	19.615	16.341

One of the most used measures of model quality is the Akaike Information Criterion (AIC). It is a complexity penalty based on information theory. The AIC considers how well the model fits the data and how many parameters were adjusted to get that result. The idea behind AIC is to choose a model with the fewest number of parameters that nevertheless adequately fits the data, preferring simpler and more parsimonious models. The ARDL short and long-term estimations are shown in Table 5.

**Table 5:** ARDL short- and long-term estimates.

Dependent Variable: SMM				
ARDL(1, 1, 1, 1, 0, 0, 1)				
Sample: 1990Q1 2022Q4				
**Variables**	**Coefficient**	**Std. Error**	**t-Statistic**	**Prob.**
**Short Run Coefficients**				
D(FINTECH)	0.017	0.005	3.195	0.001
D(ISMT)	−0.122	0.016	−7.476	0.000
D(GSFTM)	−0.294	0.063	−4.671	0.000
D(ENVCOMP)	−0.023	0.011	−2.008	0.046
D(SLO)	−0.165	0.115	−1.431	0.155
D(TIR)	0.049	0.010	4.640	0.000
CointEq(-1)	−0.205	0.053	−3.814	0.000
**Long Run Coefficients**				
FINTECH	0.011	0.010	1.061	0.290
ISMT	−0.115	0.035	−3.282	0.001
GSFTM	−0.213	0.099	−2.137	0.034
ENVCOMP	−0.113	0.046	−2.421	0.017
SLO	−0.806	0.560	−1.438	0.152
TIR	0.035	0.011	3.225	0.001
C	18.951	7.162	2.645	0.009
**Diagnostic Test Estimates**				
LM Test	0.875 (0.419)	F-statistics	241.012 (0.000)	
ARCH Test	0.050 (0.822)	Durbin-Watson	1.850	

The results reveal FINTECH's significant and positive impact on SMM in the short run. The result indicates that an increase in financial technology adoption, as reflected by more internet users, is associated with a corresponding rise in the mineral depletion rate. One plausible economic reason behind this finding is that financial technology adoption enhances the efficiency and accessibility of financial services and transactions in the mining sector. As the mining industry involves various financial operations, including funding, investment, and revenue management, adopting financial technology can streamline these processes, leading to better financial management and resource allocation [60]. Digital payment systems and online banking services can streamline the mining industry's financial activities, lowering transaction costs and boosting overall operational efficiency. In addition, financial technology may play a significant role in easing cash flow and encouraging investment in the mining sector [61]. Investors can make better judgments and manage money with the help of digital platforms that provide better access to financial information and services. This may encourage more mining, which might speed up the depletion of mineral resources. However, the positive correlation between FINTECH adoption and mineral depletion rate has environmental concerns that must be considered.

As financial technology facilitates ease of doing business and potentially attracts more investments in the mining sector, there may be increased pressure on natural resources, leading to higher rates of mineral extraction [62]. This could exacerbate environmental challenges such as habitat destruction, deforestation, and increased carbon emissions associated with mining activities. Therefore, policymakers and stakeholders need to balance leveraging financial technology for the development and growth of the mining sector while ensuring sustainable and responsible resource management. Implementing stringent environmental regulations and sustainable mining practices is crucial to mitigate the negative environmental impacts of increased mineral depletion rates from financial technology adoption [63]. This can be achieved by promoting eco-friendly mining technologies, environmental compliance, and greater emphasis on reclamation and environmental restoration measures to counterbalance the environmental costs associated with increased mining activities [64].

On the other hand, ISMT, representing investment in sustainable mining technologies, exhibits a negative and highly significant impact on SMM, both in the short- and long run. This result holds important economic and environmental implications. Increased investments in sustainable mining technologies contribute to more eco-friendly and efficient mining methods, as shown by the negative effect of ISMT on the mineral depletion rate. A wide variety of inventions go under the umbrella of "sustainable mining technologies," all of which attempt to lessen the negative effects of mining on the environment, save resources, and save money. Mining businesses may improve resource efficiency, optimize extraction operations, and lessen their use of nonrenewable resources if they use sustainable mining technology [65]. A more sustainable approach to mining leads to a slower pace of mineral depletion by ensuring that resources are mined and used more responsibly and efficiently. In this way, ISMT's detrimental effect on the mineral depletion rate indicates progress toward sustainable resource management and alleviates pressure on limited mineral supplies. In addition, reducing the negative effects of mining on the environment requires the widespread use of environmentally friendly techniques [66]. Habitat loss, soil and water pollution, and other environmental problems have often been linked to conventional mining methods. Sustainable mining practices help corporations reduce their environmental impact and protect local ecosystems. Moreover, sustainable mining methods may help decrease GHG emissions, contributing to global efforts to fight climate change and promote environmental sustainability [67]. Sustainable mining technologies provide the potential for long-term financial savings and streamlined operations. Mining companies can improve their overall financial performance by reducing resource wastage and optimizing production processes. Additionally, adopting sustainable practices can enhance a company's reputation and attract socially responsible investors, improving access to capital and financial resources [68].

Similarly, GSFTM, which represents the level of government support for FinTech in the mining sector, shows a negative and statistically significant influence on SMM in the short- and long run. This implies that a stronger governmental backing for FinTech initiatives in mining is linked to a lower mineral depletion rate, possibly due to increased efficiency and environmental considerations. One plausible economic reasoning behind this finding is that government support for FinTech in mining can enhance the transparency and accountability of mining operations. Digital technologies can enable real-time data monitoring, traceability, and auditing of mining activities, ensuring that companies adhere to environmental regulations and best practices [69]. Additionally, advanced data analytics and automation in mining can lead to better resource planning and management, optimizing production processes and reducing over-extraction of minerals. FinTech projects may help mining companies shift to a circular economy [70] if they get government funding. Governments may aid in lowering the need for ongoing resource extraction and minimizing waste by encouraging the development of circular business models that include reusing, recycling, and repurposing resources. As a result of this paradigm change toward a circular economy, mineral resources may be used more sustainably and for longer periods. From a green viewpoint, GSFTM can help encourage the adoption of sustainable procedures in the mining industry. FinTech solutions may facilitate the introduction of renewable energy integration and water recycling systems, two examples of environmentally friendly mining technologies that cut down on carbon emissions and water use in the mining industry [71]. The mining sector may become more environmentally responsible and better able to deal with environmental concerns and safeguard natural ecosystems if the government provides financial and other assistance for implementing green technology.

Short-term and long-term SMM performance is negatively affected by ENVCOMP, which stands for environmental compliance as measured by CO2 emissions from manufacturing industries and construction. This demonstrates the significance of sustainable mining techniques in lowering environmental effects since a greater degree of compliance with environmental regulations is linked to a slower rate of mineral depletion. This discovery demonstrates the significance of environmental compliance in moulding long-term mining strategies. Environmental compliance is the rate at which rules and regulations are followed to lessen the negative effects of human activity on the natural environment, such as carbon dioxide emissions from factories and building sites. Compliance with environmental regulations shows how seriously a company takes its responsibility to lessen its impact on the environment via mining [72]. The economic rationale for this conclusion is that mining firms are incentivized and required to switch to cleaner and more efficient technology due to environmental compliance efforts. Cleaner energy sources, energy-efficient equipment, and enhanced waste management systems are all things that the mining sector may benefit from when authorities impose emission reduction objectives and environmental requirements [73]. This leads to less mineral depletion and more responsible mining practices by reducing the carbon emissions the mining process produces. In addition, mining firms with a stronger commitment to environmental compliance are more likely to gain the trust of environmentally aware investors and consumers. Consumers and investors alike are increasingly looking for signs that a company is committed to environmental sustainability [74] to choose whether or not to do business with them. Mining businesses may gain credibility, trust, and long-term investment by limiting their environmental effect and operating by applicable legislation. In addition, mining innovation might be fueled by environmental regulations. When faced with stricter rules, businesses typically look for new ways to lessen their effect on the environment. This may lead to developing and adopting more sustainable mining practices, such as carbon capture and storage, energy-efficient mining processes, and reclamation techniques, which can further contribute to a lower mineral depletion rate [75]. From an environmental perspective, the negative impact of ENVCOMP on the mineral depletion rate indicates that environmentally conscious policies and regulations can effectively curtail unsustainable mining practices and protect natural ecosystems. By reducing CO2 emissions and other environmental pollutants, environmental compliance measures contribute to environmental preservation and conservation of biodiversity, ensuring the long-term ecological health of the regions affected by mining activities.

**Fig. 1:**
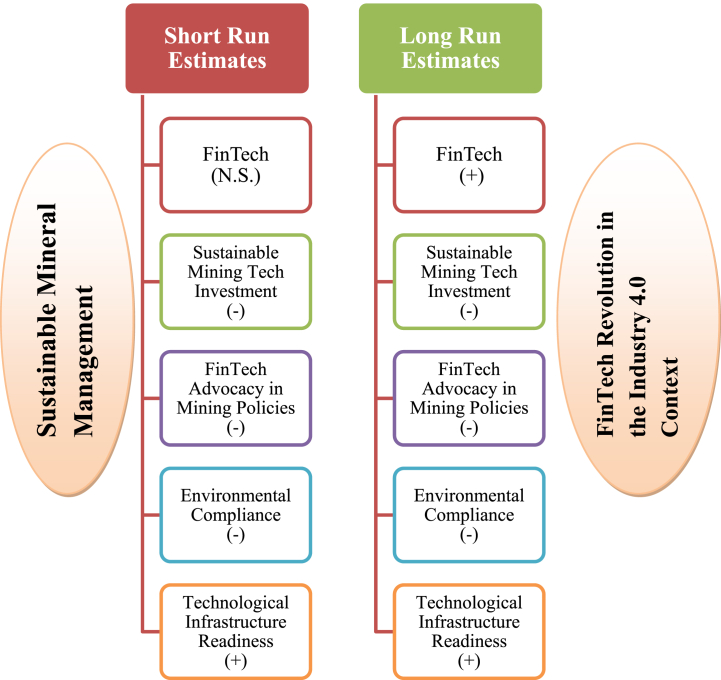
Summary of the results.

A positive relationship between TIR and SMM both in the short- and long-run suggests that improved technological infrastructure readiness, represented by fixed broadband subscriptions, is associated with a higher mineral depletion rate. Advanced technological infrastructure enhances the efficiency and productivity of mining operations. With better access to fixed broadband subscriptions, mining companies can leverage advanced technologies, data analytics, and automation to optimize their mining activities. This may include real-time monitoring of mining sites, remote-controlled machinery, and automated processes that streamline resource extraction and increase production rates [76]. As a result, the higher mineral depletion rate associated with improved TIR could indicate mining activities being conducted more swiftly and effectively due to technological advancements. Moreover, increased technological infrastructure readiness may attract more investments and exploration in the mining sector. Mining companies are more likely to invest in regions with robust technological connectivity, facilitating better communication, data sharing, and operational efficiency [77]. As exploration and mining operations expand, the mineral depletion rate is expected to rise in these areas, leading to an association between improved TIR and higher mineral depletion rates. A rise in demand for mineral resources, spurred by technical progress in other sectors, may also mitigate the negative effect of TIR on the mineral depletion rate. Increases in the availability of minerals and raw materials are frequently necessary to sustain the production of technological products and equipment as technology progresses [78]. Because of this, the pace at which minerals are being exhausted from the earth may speed up as more effort is put into mining them. From an environmental perspective, however, the positive association between TIR and the mineral depletion rate raises concerns about the potential environmental impacts of increased mining activities. Higher mining rates could strain natural ecosystems more, lead to habitat destruction, and exacerbate environmental degradation [79]. Therefore, it is essential to balance the benefits of improved technological infrastructure with the need for responsible and sustainable mining practices to mitigate the adverse environmental effects. Fig. 1 provides a snapshot of the results, emphasizing the most critical takeaways in terms of long-term resource management.

Source; Author's estimate.

In the given table, the error correction term value of −0.205 with a t-value of −3.814 indicates the speed of adjustment towards the long-run equilibrium relationship between the variables in the model. A significant negative error correction term suggests the model has a stable and meaningful long-term relationship between the variables. When short-term shocks or deviations occur, the system will tend to correct them over time, bringing the variables back to their long-run equilibrium. This adjustment process is essential in economic analysis as it helps to ensure that the relationships established between the variables are not spurious but have a stable and meaningful long-term connection. Table 6 shows the Granger causality estimates.

**Table 6:** Granger causality estimates.

Unidirectional Causality	Decision	Bidirectional Causality	Decision
FINTECH → SMM	Financial technology –led sustainable mineral management and technological infrastructure readiness.	GSFTM ↔ FINTECH	Dynamic and mutually influential relationship between government support for FinTech in mining and the adoption of financial technology in the sector
FINTECH → TIR			
ENVCOMP → SLO	Environmental compliance –led social license to operate mining and technological readiness.	GSFTM ↔ SMM	Interconnectedness of policy interventions, technological advancements, and environmental considerations in the mining sector.
ENVCOMP → TIR			
TIR → SLO	Technology readiness –led social license to operate mining.		
GSFTM → TIR	Government support for FinTech in mining –led technology readiness.		

The results show that financial technology (FinTech) leads to sustainable mineral management through improved technological infrastructure readiness. Environmental compliance drives the social license to operate in mining and enhances technological readiness. Technology readiness fosters a social license to operate in mining. Government support for FinTech in mining promotes technology readiness in the sector. Bidirectional causality between GSFTM and FINTECH implies that the two variables influence each other, creating a feedback loop or a dynamic relationship. When there is a positive change in GSFTM, it can lead to an increase in FINTECH adoption in the mining sector. On the other hand, when there is an increase in FINTECH adoption, it can influence the level of government support provided for FinTech initiatives in the mining industry. This symbiotic link between government policies and technology adoption in the mining industry and the GSFTM and FINTECH as a mutual causation might indicate this. Increased technology innovations and efficiency in the mining industry are possible when the government establishes supportive regulations and offers financial incentives to stimulate FinTech use. These technological developments might persuade policymakers to invest even more in the sector, setting off a virtuous cycle of ever-increasing quality of life. Given the two-way nature of the relationship between GSFTM and SMM, it stands to reason that sustainable mineral management can affect the extent to which governments back FinTech initiatives in the mining industry and vice versa. This two-way causation may have economic justification since government encouragement of FinTech in mining may boost innovation and efficiency in the industry. The adoption of financial technology in mining operations may be aided by government policies that encourage its use and give financial incentives and regulatory assistance. This might eventually lead to more sustainable mineral management via more efficient and ecologically friendly mining processes. Government support for FinTech in mining may also be affected by the prevalence of sustainable mineral management methods. The mining industry's image and credibility as a caretaker of the environment might benefit from greater efforts to ensure the long-term viability of its resources. In response, the government might be more inclined to support FinTech initiatives to promote sustainable mining practices and technological advancements further. Table 7 shows the IRF estimates of SMM.

**Table 7:** IRF estimates of SMM.

Years	SMM	FINTECH	ISMT	GSFTM	ENVCOMP	SLO	TIR
2023	0.169	0	0	0	0	0	0
2024	0.158	−0.002	−0.003	−0.002	−0.002	−0.003	0.001
2025	0.137	−0.0002	−0.005	−0.001	−0.006	−0.009	0.005
2026	0.118	0.0008	−0.006	−0.0004	−0.009	−0.013	0.007
2027	0.103	0.002	−0.006	0.0006	−0.010	−0.014	0.008
2028	0.091	0.003	−0.005	0.001	−0.011	−0.014	0.009
2029	0.081	0.004	−0.004	0.002	−0.011	−0.013	0.010
2030	0.072	0.005	−0.003	0.003	−0.011	−0.012	0.010
2031	0.064	0.005	−0.002	0.003	−0.011	−0.010	0.010
2032	0.058	0.006	−0.001	0.004	−0.010	−0.009	0.010

The results show that FinTech, GSFTM, and TIR will positively impact SMM over the next ten years. This suggests that adopting financial technology, increased government support for FinTech initiatives in mining, and improved technological infrastructure readiness will contribute positively to the mineral depletion rate and sustainable mining practices during the coming decade. On the other hand, ISMT, ENVCOMP, and SLO are likely to have a negative impact on SMM for the next ten years. This implies that higher investments in sustainable mining technologies, stricter environmental compliance measures, and social license issues might result in a decrease in the mineral depletion rate or a more cautious approach to mining activities during the specified period. These projected impacts highlight the significance of various factors in shaping the future of sustainable mineral management. Table 8 shows the VDA estimates of SMM.

**Table 8:** VDA estimates of SMM.

Years	S.E.	SMM	FINTECH	ISMT	GSFTM	ENVCOMP	SLO	TIR
2023	0.169	100	0	0	0	0	0	0
2024	0.232	99.911	0.012	0.019	0.015	0.013	0.022	0.005
2025	0.270	99.678	0.009	0.052	0.013	0.071	0.135	0.039
2026	0.295	99.326	0.008	0.088	0.011	0.161	0.309	0.095
2027	0.313	98.945	0.011	0.117	0.010	0.263	0.485	0.165
2028	0.327	98.584	0.020	0.138	0.011	0.366	0.635	0.243
2029	0.338	98.260	0.034	0.150	0.015	0.462	0.752	0.324
2030	0.346	97.971	0.053	0.155	0.022	0.551	0.840	0.404
2031	0.353	97.711	0.079	0.156	0.033	0.632	0.903	0.483
2032	0.358	97.471	0.111	0.153	0.048	0.707	0.949	0.558

According to the results, ISMT is projected to have the greatest variance shock on SMM over the next ten years, with a percentage of 0.153 %. This indicates that fluctuations or changes in investment levels in sustainable mining technologies will significantly impact the mineral depletion rate during the specified time period. Following ISMT, the next highest variance shock on SMM is expected to come from FINTECH at 0.111 %. This implies that variations in the adoption of financial technology, represented by changes in the number of internet users, will also have a notable effect on the sustainable mineral management proxy during the coming decade. SLO (Social License to Operate) is projected to have the third-highest variance shock on SMM at 0.949 %. This suggests that social license considerations, such as community acceptance and support, will substantially influence the mineral depletion rate over the next ten years. ENVCOMP is anticipated to have a variance shock of 0.707 % on SMM, indicating that fluctuations in environmental compliance, particularly related to CO2 emissions from manufacturing industries and construction, will significantly affect sustainable mineral management practices. Finally, GSFTM is projected to have the smallest variance shock on SMM, at 0.048 %. This suggests that changes in government support for FinTech initiatives in the mining sector, as measured by the ease of doing business score, will have a relatively minor impact on the mineral depletion rate during the specified 10-year period.

## 5 Conclusions and policy implications

The study provides valuable insights into the influence of financial technology (FinTech) adoption on implementing sustainable mineral management policies in Australia. The findings indicate that FinTech adoption and technological infrastructure readiness exhibit a positive association with eco-conscious mining policies in the short run. However, investment in sustainable mining technologies, government support for FinTech in mining, and environmental compliance demonstrate a negative relationship with responsible resource management in the short and long run. The bidirectional causality between regulatory support for mining FinTech and technology-enabled financial services, as well as environmentally conscious mineral practices, underscores the interconnectedness between policy interventions and technological advancements in the mining sector. Moreover, the unidirectional causality from FinTech adoption to sustainable mining practices and technology preparedness highlights the potential of financial technology to drive advancements in eco-friendly resource management. These findings have significant implications for policymakers and industry stakeholders, emphasizing the importance of balancing technological innovation and sustainable resource utilization. By fostering a conducive environment for FinTech adoption and promoting investments in sustainable mining technologies, policymakers can pave the way for responsible mining practices in the context of Industry 4.0. The Australian economy can benefit from the following suggested policy implications for sustainable mineral resource management:

- Short-Term Policy Implications
  1. Encourage the adoption of financial technology across the mining sector to enhance operational efficiency, resource allocation, and environmental monitoring in the short term.
  2. Implement stringent environmental regulations and compliance standards to ensure responsible mining practices and minimize environmental impacts.
  3. Facilitate investments in technological infrastructure readiness, such as broadband connectivity, to enable seamless data exchange and leverage advanced technologies for sustainable mineral management.
  4. Provide incentives for research and development in sustainable mining technologies, fostering innovation and sustainable practices within the industry.
  5. Foster transparent and open communication with local communities and stakeholders to obtain social acceptance and build a strong social license to operate in the short term.
- Medium-Term Policy Implications:
  1. Introduce financial incentives for mining companies adopting eco-friendly practices and technologies, encouraging long-term sustainability in the medium term.
  2. Implement policies that promote circular economy principles within the mining industry, emphasizing resource recycling and minimizing waste generation.
  3. Invest in training programs to use advanced technologies and environmentally conscious mining practices to upskill the mining workforce.
  4. Encourage partnerships between the government, industry stakeholders, and research institutions to address sustainability challenges in the medium term collaboratively.
  5. Establish certification standards for environmentally responsible mining practices, providing market differentiation for companies adhering to sustainable principles.
- Long-Term Policy Implications:
  1. Integrate sustainability principles into overarching mining policies, ensuring a long-term focus on responsible resource management.
  2. Encourage green financing mechanisms to fund sustainable mining projects and incentivize eco-conscious investments.
  3. Establish innovation hubs and centers of excellence for sustainable mining technologies, driving long-term technological advancements.
  4. Implement mandatory environmental, social, and governance (ESG) reporting for mining companies to enhance transparency and accountability in the long term.
  5. Develop a comprehensive roadmap for transitioning the mining industry towards a circular economy model, minimizing resource extraction and promoting resource reuse and recycling in the long term.

These policy implications guide policymakers and industry stakeholders to navigate the Australian economy towards a more sustainable and responsible mining sector.

### 5.1 Study's limitations and future directions

The study's relevance outside Australia is wavering, and its temporal limitations may obscure subtleties over the long run. There may be unknown issues due to data constraints and a narrow emphasis on certain variables. Future studies might entail cross-national comparisons, undertake longitudinal studies, integrate qualitative data, examine other factors, and analyze the actual effect of suggested reforms. These actions would help us learn more about the connections between the use of FinTech, technological preparedness, and responsible mineral management.

## CRediT authorship contribution statement

**Yuanyuan Xu:** Writing – review & editing, Writing – original draft, Validation, Resources, Project administration, Methodology, Investigation, Funding acquisition, Formal analysis, Data curation, Conceptualization. **Abdelmohsen A. Nassani:** Writing – review & editing, Writing – original draft, Supervision, Resources, Project administration, Methodology, Investigation, Funding acquisition, Formal analysis, Data curation, Conceptualization. **Muhammad Moinuddin Qazi Abro:** Writing – review & editing, Writing – original draft, Visualization, Validation, Resources, Project administration, Methodology, Investigation, Formal analysis, Data curation, Conceptualization. **Imran Naseem:** Writing – review & editing, Writing – original draft, Visualization, Validation, Resources, Project administration, Methodology, Investigation, Formal analysis, Data curation, Conceptualization. **Khalid Zaman:** Writing – original draft, Validation, Software, Methodology, Funding acquisition, Formal analysis, Conceptualization.

## Declaration of competing interest

The authors declare that they have no known competing financial interests or personal relationships that could have appeared to influence the work reported in this paper.
